# Metatranscriptomic Assessment of the Microbial Community Associated With the Flavescence dorée Phytoplasma Insect Vector *Scaphoideus titanus*

**DOI:** 10.3389/fmicb.2022.866523

**Published:** 2022-04-19

**Authors:** Simona Abbà, Marika Rossi, Marta Vallino, Luciana Galetto, Cristina Marzachì, Massimo Turina

**Affiliations:** Consiglio Nazionale delle Ricerche-Istituto per la Protezione Sostenibile delle Piante (CNR-IPSP), Turin, Italy

**Keywords:** microbiome, phytoplasma, insect vector, metatranscriptomics, *Candidatus* Sulcia muelleri, primary symbionts, *Ophiocordyceps*-allied fungus

## Abstract

Phytoplasmas are insect-borne pathogenic bacteria that cause major economic losses to several crops worldwide. The dynamic microbial community associated with insect vectors influences several aspects of their biology, including their vector competence for pathogens. Unraveling the diversity of the microbiome of phytoplasma insect vectors is gaining increasing importance in the quest to develop novel microbe-based pest control strategies that can minimize the use of insecticides for better environmental quality. The leafhopper *Scaphoideus titanus* is the primary vector of the Flavescence dorée phytoplasma, a quarantine pest which is dramatically affecting the main grape-growing European countries. In this study, the RNA-Seq data, which were previously used for insect virus discovery, were further explored to assess the composition of the whole microbial community associated with insects caught in the wild in both its native (the United States) and invasive (Europe) areas. The first *de novo* assembly of the insect transcriptome was used to filter the host sequencing reads. The remaining ones were assembled into contigs and analyzed by blastx to provide the taxonomic identification of the microorganisms associated with *S. titanus*, including the non-bacterial components. By comparing the transcriptomic libraries, we could differentiate the stable and consistent associations from the more ephemeral and flexible ones. Two species appeared to be universal to the core microbiome of *S. titanus:* the obligate bacterial symbiont *Candidatus* Sulcia muelleri and an *Ophiocordyceps*-allied fungus distantly related to yeast-like symbionts described from other hemipterans. Bacteria of the genus *Cardinium* have been identified as another dominant member of the microbiome, but only in the European specimens. Although we are yet to witness how the interplay among the microorganisms influences the vector competence of *S. titanus*, this unbiased *in silico* characterization of its microbiome is paramount for identifying the naturally occurring targets for new biocontrol strategies to counteract Flavescence dorée spread in Europe.

## Introduction

Insects live in close association with multiple microorganisms that can profoundly affect several aspects of their biology: from their supplies for essential nutrients to their responses against biotic or abiotic stressors, from inter- and intraspecific communication to their vectoring efficiency (Engel and Moran, 2013). The dynamic microbial community occupying the host-associated niche is collectively referred to as the microbiome (Whipps and Karen Lewis, 1988), which, according to a recent extension of the definition, also includes microbial structures, metabolites, mobile genetic elements (e.g., transposons, phages, and viruses), and relic DNA (Berg et al., 2020).

The idea that insects form an indissoluble unit function with their associated microbes, the so-called holobiont, is behind the efforts to identify microorganisms that could be either selectively eliminated from the microbiome or, on the contrary, introduced with or without previous genetic manipulation to negatively impact the fitness and/or the transmission competence of insect vectors. Hence, characterizing the diversity of symbionts is the first step in the current quest to develop novel microbe-based vector-control strategies. The ultimate goal is the development and application of biocontrol strategies that can minimize the use of insecticides and meet the demands of public opinion for better environmental quality.

The majority of studies about microbiomes of insect vectors have been conducted so far on medically important mosquito genera (recently reviewed by Cansado-Utrilla et al., 2021). The observation that *Wolbachia* inhibits the replication of various mosquito-borne, human-pathogenic viruses (Moreira et al., 2009) led, for example, to the successful development of *Wolbachia*-based strategies to control the viruses transmitted by *Aedes aegypti* in several tropical areas, as part of the World Mosquito Program (reviewed by O'Neill, 2018). The past decade has also witnessed a rise in the studies describing microbiome dynamics in insect vectors of plant pathogens, such as whiteflies (Himler et al., 2011; Su et al., 2015), psyllids (Fagen, 2012; Meng et al., 2019), sharpshooters (Hail et al., 2011; Rogers and Backus, 2014) and aphids (McLean et al., 2019; He et al., 2021). Likewise, unraveling the diversity of microorganisms associated with phytoplasma insect vectors is gaining increasing importance due to the devastating impact of phytoplasmas on agricultural production worldwide (Kumari et al., 2019) and the recent finding that their diversity and potential insect host range have been so far largely underestimated (Trivellone et al., 2021). Insect vectors of these bacterial plant pathogens are phloem feeders of the order *Hemiptera*, mainly leafhoppers, planthoppers, and psyllids. *Scaphoideus titanus* Ball is a phloem-feeding Nearctic leafhopper that was probably introduced accidentally from North America to Europe (Papura et al., 2012). It is the primary vector of Flavescence dorée phytoplasma (FDp), a quarantine pest currently present in the main grape-growing European countries (EFSA Panel on Plant Health et al., 2016). Most of the knowledge about the microbiome associated with *S. titanus* and other phytoplasma vectors is currently based on laboratory-reared insects and focuses almost exclusively on a few bacterial components (Campbell and Purcell, 1993; Marzorati et al., 2006; Sacchi et al., 2008; Gonella et al., 2011; Wangkeeree et al., 2012; Ishii et al., 2013; Powell et al., 2015; Iasur-Kruh et al., 2018). Only recently, a 16S metabarcoding analysis was conducted to provide a broader view of the bacterial microorganisms associated with “*Candidatus* Phytoplasma solani” infected and non-infected insects caught in nature (Moussa et al., 2020), but such an approach neglects the other components of the microbiome that might be associated with the phytoplasma insect vectors (e.g., protozoa, fungi, and viruses).

Here, an unbiased metatranscriptomic approach revealed the previously underestimated complexity of the transcriptionally active microbial communities associated with field-collected *S. titanus* populations. The RNA-Seq data previously used for virus discovery (Ottati et al., 2020) were re-analyzed in this work to generate the *de novo* transcriptome assembly of the insect host as well as to expand our understanding of the breadth of its microbial diversity both in its invasive (Europe) and native (United States) geographical areas. In addition to the taxonomic identification of the microbial components, we defined *S. titanus* core microbiome, which appeared to be dominated by the obligate endosymbiont *Candidatus* Sulcia muelleri and an *Ophiocordyceps*-allied fungus distantly related to symbiotic fungi described from other hemipterans. We also discussed the bioinformatic challenges related to the investigation of the microbial communities associated with a non-model organism such as *S. titanus*, for which only a very limited amount of sequence information is available.

## Materials and Methods

### Insect Collection, RNA Preparation, and Sequencing

Detailed information about insect populations, RNA extraction, library preparation, and sequencing can be found in the paper by Ottati et al. (2020). Briefly, *S. titanus* specimens were collected from the field with a sweep net in 15 European sites and one American site during the summer of 2018. The RNA extracts were pooled for library construction according to the country of origin of the insects: St_FR library for France, St_CH for Switzerland, St_HU for Hungary, St_USA for the United States of America, and St_IT1 and St_IT2 for the Italian samples. Ribosomal RNA-depleted libraries were prepared using a TruSeq Stranded Total RNA Library Prep Kit (v2) (Illumina Inc. San Diego, CA, United States) and were sequenced from both ends (100 bp) using the NovaSeq System by Macrogen Inc. (Seoul, South Korea).

### Bioinformatic Analysis

The workflow for *S. titanus* metatranscriptomic analysis was based on the approach used by Batson and colleagues (Batson et al., 2021) for the characterization of mosquito metatranscriptomes.

Raw paired-end files were processed for the removal of Illumina adaptor sequences and artifacts, for length and ribosomal filtering, and for the removal of reads matching unwanted environmental contaminants (human, mouse, cat, and dog sequences) using the suite of bioinformatic tools called (BBtools, 2021) v38.70 (Sourceforge.net/projects/bbmap/). The St_IT1 library was used as a reference for the analysis of the other five. After normalizing read coverage with BBnorm, the transcriptome assembly was performed with Trinity v2.9.1 (Grabherr et al., 2011). The reads used for the assembly were mapped back to the contigs using Bowtie2 (Langmead and Salzberg, 2012) (flags:–very-sensitive –no-mixed –no-discordant), and only contigs with a mapping index ≥1 were retained for further analysis. The mapping index was defined as (number of mapped reads ^*^ 100)/contig length), where 100 represents the average read length. This method selected only those contigs whose length was at least equal to the sum of the lengths of the mapping reads. As far as the analysis of the dark matter was concerned, SAMtools (Li et al., 2009) separated the reads that mapped on sense and antisense RNA strands.

Sequences with index ≥1 were clustered with CD-HIT (Li and Godzik, 2006) with a threshold of ≥ 90% identity to reduce redundancy. DIAMOND v0.9.24.125 (Buchfink et al., 2015) was used to perform a local blastx search against the NCBI non-redundant protein database (released on August 14, 2021) formatted to associate every sequence with a taxonomic node through the NCBI TaxID (unique identifiers for each taxon).

For each assembled contig, a maximum of five hits with an *E* ≤ 0.0001 was retrieved, if available. Then, two custom-made Perl scripts were run to: (1) assign each contig to a phylum on the basis of the phyla of the first five blastx hits and (2) if a phylum was assigned, associate the contig to the function of the first best hit belonging to that phylum. The contigs were defined as taxonomically “ambiguous” if (a) the blastx algorithm retrieved less than three significant hits or (b) there were at least three significant blastx hits, but none of the phyla assigned to these hits was predominant (at least 66%) over the others, for example, an assembled contig found significant matches with three sequences assigned to *Arthropoda* and two sequences assigned to *Proteobacteria*.

The contigs assigned to *Arthropoda* were considered as part of *S. titanus* transcriptome. All the other contigs with a taxon assignment were considered as non-host contigs and were used to describe the *S. titanus* microbiome composition.

Once an *S. titanus* reference transcriptome was defined from the St_IT1 library, the other five libraries were analyzed in the same way as previously described, but with the addition of a further step, which consisted in discarding all reads that mapped to *S. titanus* transcriptome before the assembly step with Trinity. Such additional steps are aimed at simplifying the disentanglement of the multiple microbiome components.

Each taxonomically assigned contig was also aligned to the NCBI nt database (release October 2021) using blastn to exclude sequences with matches to prokaryotic or eukaryotic rRNAs (*E* ≤ 1e-20) or to the most common environmental contaminants, for example, human, dog, cat, and mouse sequences. Although the libraries were ribosomal RNA (rRNA)-depleted and the read quality filtering included a step for the removal of the remaining ribosomal reads, the assembled contigs with significant matches to rRNAs were, in fact, still present in the datasets.

The putative non-host proteins were identified with TransDecoder (2021) v5.5.0 (https://github.com/TransDecoder/TransDecoder) and further characterized by the eggNOG-Mapper (Cantalapiedra et al., 2021) and a Batch CD-Search against the NCBI Conserved Domain Database (CDD) (Lu et al., 2020), using default parameters.

The quantitative assessment of *S. titanus* transcriptome completeness in terms of the expected gene content was evaluated using BUSCO 5.0 (Simão et al., 2015) against the *Hemiptera* ortholog list in OrthoDB v10.

### Phylogenetic Tree

Phylogenetic relationships were inferred using the fungal 18S rRNA sequences provided by Matsuura et al. (2018). Multiple alignments of the nucleotide sequences were generated using the program MAFFT (v. 7.271) (Katoh and Standley, 2013). JModelTest2 v2.1.10 (Darriba et al., 2012) was used to find the preferred model of evolution to analyze the sequences. The phylogenetic tree was inferred by MrBayes v3.2.7 (Ronquist et al., 2012) with the Kimura 2-parameters (K2P) + Γ + I model. The CIPRES Science Gateway V 3.3 (Miller et al., 2010) was used to perform MrBayes analysis with the following settings: GTR + invgamma; nst = 2; statefreqpr=fixed(equal), 10^7^ generations, sampling every 1,000 generations with a burnin fraction of 0.25.

### Read Mapping and Treemaps

According to the criteria described in the previous paragraph, the non-host reads were assigned to the taxonomic group of the contig they were mapped to. The counts were normalized according to the total of quality-filtered reads for each library, instead of the total of non-host reads, because the number of non-host reads varied tremendously across libraries. In addition, such normalization allowed comparing the proportion of viruses, bacteria, and eukaryotic reads with the amount of *S. titanus*-assigned or ambiguous reads. Such an approach was intended to provide a quantification of the impact of each microbial component to the transcriptional profile of the holobiont, promoting the identification of the microorganisms that might cause the most dramatic consequences on the host, once targeted for biocontrol purposes.

The distribution of reads among the phyla was represented by treemaps drawn with the R package treemap (version 2.4–3) as nested rectangles whose area is proportional to the numerical values. Datasets are structured in a hierarchical order so that it is possible to quickly identify the larger and smaller components within a given library. All phyla with a minimum of 1,000 reads in at least one of the six libraries were reported in tables and treemaps (Supplementary Image 1), whereas the others (e.g., *Cyanobacteria, Basidiomycota, Chlorophyta, Microsporidia*, etc.) converged in a generic category named “Others.” An additional category named “Unclassified viruses” was introduced to accommodate the RNA viruses without a taxonomic lineage.

### Data Availability

Raw (SRA accessions: SRR16109749-SRR16109754) and assembled sequencing data (TSA accessions: GJQL00000000, GJQM00000000, GJQN00000000, GJQO00000000, GJQP00000000, GJQ00000000) are deposited in the NCBI Bioproject PRJNA765507. Segment 12 of Scaphoideus titanus reo-like virus 1 and the partial sequence of the 18S rRNA gene of *Scaphoideus titanus* yeast-like symbiont are deposited in the GenBank database under the accession numbers OM103795 and OM311272, respectively.

## Results

### Relative Abundance of Host, Non-host, Ambiguous, and Unknown Reads

To characterize the microbial cargo of *S. titanus* for each library, we first calculated the overall proportion of the reads assembled into contigs that could be assigned to the phylum *Arthropoda* out of the total number of quality-filtered reads (Table 1).

**Table 1:** Percentages of the host (*Arthropoda*), non-host (other phyla), ambiguous, and unknown (dark matter) reads in each library.

	**St_IT1**	**St_IT2**	**St_FR**	**St_CH**	**St_HU**	**St_USA**
*Arthropoda*	85.9	89.7	75.8	87.6	85.5	50.8
Other phyla	3.0	3.3	2.0	2.1	1.5	19.2 (viruses = 18.5)
Ambiguous	5.8	3.6	1.6	3.1	2.4	4.0
Dark matter	5.2	3.4	20.5	7.2	10.5	26.0
Quality filtered reads	27,323,556	25,392,886	23,102,054	28,095,006	27,686,890	33,105,154

In the St_IT1 library, a total of 27,103 transcripts (N50 sequence length: 2,879 nt) were assembled and assigned to the members of the phylum *Arthropoda* (Supplementary Data Sheet 1). In the absence of the *S. titanus* genome to verify blastx predictions, those sequences were considered insect transcripts. All those transcripts, including putative isoforms and paralogs assembled by the Trinity software, were used as a reference for the BUSCO assessment of transcriptome completeness, with the following results: the assembly was 94.6% complete, with 2.5% fragmentation, 10.1% duplication, and 5.4% of missing core genes. The reads mapping to those *S. titanus* transcripts represented at least three-quarters of all reads in each library, except the St_USA library, where they represented only 50%. The St_USA library diverged from the European ones also for the highest percentage of reads assigned to members of the phyla other than *Arthropoda* (19.2%), mainly to viral phyla. In all libraries, less than 6% of reads could not be unambiguously assigned to a specific phylum, even if they mapped to transcripts with significant similarities to sequences deposited in the NCBI nr database (“ambiguous” in Table 1). By subtraction from a minimum of 3.4% (St_IT2 library) to a maximum of 26.0% (St_USA library) of all filtered reads represented, the so-called transcriptional “dark matter,” which included reads that mapped to either long non-coding RNAs or protein-coding transcripts without any recognizable sequence similarity to previously published sequences.

### A Comprehensive View of Non-host Reads Assigned to the Viral, Bacterial, and Eukaryotic Taxa

Supplementary Image 1 and Supplementary Image 2A provide for each library a quantitative treemap overview (absolute quantities) and the percentages of how the assembled reads mapped across the non-*Arthropoda* taxa, respectively. Supplementary Data Sheet 2 reports all the underlying data, blastx results, and sequences discussed in the following paragraphs. The details of the breakdown of the assignment across the phyla, normalized according to the total of quality-filtered reads for each library, are provided in Table 2. To avoid the use of percentages with many digits after the decimal point and make the data easier to read, the figures are hereafter expressed as the number of reads assigned to a phylum per 10 thousand clean reads, RPTT (e.g., 0.02% corresponds to 2 RPTT).

**Table 2:** Reads assigned to phyla other than *Arthropoda* expressed as RPTT.

	**St_IT1**	**St_IT2**	**St_FR**	**St_CH**	**St_HU**	**St_USA**
**Bacteria**	**182.9**	**170.4**	**187.5**	**193.9**	**140.5**	**50.5**
*Bacteroidetes^§^*	178.3 (S = 158.6; C = 19.6)	167.8 (S = 146.3; C = 21.5)	181.5 (S = 171.8; C = 9.5)	190.5 (S = 173.4; C = 17.0)	137.9 (S = 132.5; C = 5.4)	45.4 (S = 45.4)
*Proteobacteria*	2.1	1.6	2.3	1.9	1.6	4.4
*Firmicutes*	1.6	1.0	3.7	1.5	1.0	0.7
*Tenericutes*	0.9	0.0	0.0	0.0	0.0	0.0
**Fungi**	**67.7**	**70.6**	**1.0**	**3.9**	**3.6**	**1.4**
*Ascomycota^§^*	67.7 (O = 40.5)	70.6 (O = 36.0)	1.0 (O = 0.7)	3.9 (O = 2.7)	3.6 (O = 2.4)	1.4 (O = 0.2)
**Non fungal eukaryotes**	**43.8**	**84.6**	**3.1**	**3.9**	**6.2**	**15**
*Annelida*	0.5	0.2	0.0	0.2	0.1	0.0
*Chordata*	22.5	24.5	1.1	1.6	2.1	7.5
*Cnidaria*	6.9	4.8	0.2	0.5	0.5	1.0
*Echinodermata*	0.5	1.4	0.0	0.0	0.1	1.7
*Mollusca*	5.3	2.0	0.6	0.4	0.6	1.6
*Nematoda*	1.3	48.5	0.1	0.5	0.3	1.6
*Rotifera*	5.6	2.6	1.0	0.5	0.6	1.0
*Streptophyta*	1.2	0.6	0.1	0.2	1.9	0.6
**Viruses**	**4.0**	**2.2**	**9.7**	**9.7**	**0.7**	**1853.8**
*Duplornaviricota*	0.5	0.5	5.7	6.6	0.1	1550.9
*Kitrinoviricota*	0.1	0.2	0.0	2.4	0.0	0.0
*Negarnaviricota*	0.4	0.8	0.4	0.5	0.4	167.7
*Pisuviricota*	2.3	0.0	1.2	0.0	0.0	9.5
*Uroviricota*	0.7	0.7	2.4	0.2	0.2	0.0
Unclassified virus	0.0	0.0	0.0	0.0	0.0	125.7
**Others**	**0.8**	**0.6**	**0.3**	**0.3**	**0.3**	**0.4**

*Reads are grouped into five main categories (bacteria, fungi, non-fungal eukaryotes, viruses, and others) indicated in bold letters. The first four categories are followed by the phyla (in italics) with a minimum of 1,000 reads in at least one of the six libraries. The category “Others” is represented by the sum of reads assigned to the phyla with less than 1,000 reads in all six libraries.*

§ *Reads assigned to Ophiocordycipitaceae (O), Candidatus Sulcia muelleri (S) and Cardinium (C) were reported in brackets*.

### *Bacteroidetes* and Insect Viruses Are the Most Transcriptionally Active Components of the *S. titanus* Microbiome

As indicated by the values reported in Table 2, the main features of the *S. titanus* microbiome that differentiated the American library from the European ones were the bacterial and viral components (Supplementary Images 2A–C). In the St_USA library reads that mapped to transcripts assigned to *Bacteroidetes* were, in fact, nearly four times less abundant than those in the other libraries. In the European ones, almost all *Bacteroidetes* reads belonged to two taxa, *Cardinium* sp. and *Candidatus* Sulcia muelleri. Interestingly, in the St_USA library, *Cardinium* was absent and the reads assigned to *Ca*. Sulcia muelleri accounted for all the *Bacteroidetes* reads. Prokaryotes were also represented by the phyla *Proteobacteria, Firmicutes*, and *Tenericutes* at much lower abundance compared to *Bacteroidetes*. The phylum *Tenericutes*, in particular, was only identified in the St_IT1 library, where it was entirely represented by the reads that mapped to sequences assigned to the phytoplasmas, mostly *Candidatus* Phytoplasma ziziphi and FDp, both members of taxonomic group 16SrV. Unlike *Bacteroidetes*, the phylum *Proteobacteria* encompassed a great variety of genera in all libraries, with the class *Gammaproteobacteria* being the most transcriptionally active taxon. *Alphaproteobacteria*, including the genera *Wolbachia* and *Rickettsia*, were present at much lower abundance.

Regarding viruses, in the St_USA library, the number of viral reads was more than 150 times higher than those identified in the European libraries (Table 2 and Supplementary Images 2A–C). The vast majority of the non-host reads that assembled into contigs in the St_USA library corresponded to the complete viral genomes identified in a previous work by Ottati et al. (2020): Scaphoideus titanus permutotetra-like virus 1 (no assigned phylum, “Unclassified virus” in Table 2), Scaphoideus titanus reo-like virus 1 (*Duplornaviricota*), Scaphoideus titanus bunya-like virus 1 (*Negarnaviricota*), Scaphoideus titanus sobemo-like virus 1, Scaphoideus titanus sobemo-like virus 2, Scaphoideus titanus iflavirus 2 and Scaphoideus titanus-associated partiti-like virus 1 (all *Pisuviricota*). Considering the European libraries, the viral reads reached a peak in the St_FR library, where both Scaphoideus titanus toti-like virus 1 (*Duplornaviricota*) and Scaphoideus titanus iflavirus 1 (*Pisuviricota*) were identified. Lower amounts of viral reads were found in St_IT2, St_IT1, and St_CH libraries, which were infected by only one of the two above-mentioned viruses. The remaining viral reads mapped to few bacteriophage transcripts (*Uroviricota*) and some endogenous viral elements, mostly related to the phylum *Negarnaviricota* (Ottati et al., 2020).

### Eukaryotic Taxa and Their Associated Viruses

The amount of reads mapping to the *Ascomycota* transcripts varied dramatically across libraries: from 10% of the total reads assigned to the eukaryotic taxa in the St_USA library to as high as 60% in the St_IT1 library (Supplementary Image 2D). The *Ophiocordycipitaceae* family (class *Sordariomycetes*, order *Hypocreales*) was detected in all samples and represented the most dominant taxon in the European libraries. This ascomycetous group consists of both the entomopathogenic fungi (Sanjuan et al., 2015; Araújo and Hughes, 2016) and *Ophiocordyceps*-allied symbionts of insects (Suh et al., 2001; Gomez-Polo et al., 2017; Matsuura et al., 2018). The second most abundant fungal taxon detected in the Italian libraries was the family *Erysiphaceae*, whose species live epiphytically on the outer surface of plants.

The Zooplankton species, namely organisms of the aquatic food chain belonging to the phyla *Annelida, Cnidaria, Rotifera, Mollusca*, and *Echinodermata*, overall accounted for a maximum of 19.1 RPTT in the St_IT1 library to a minimum of 1.7 RPTT in the St_CH library.

The reads assigned to *Nematoda* generally represented a small proportion of the non-host reads, except in the case of the St_IT2 library, where they reached 48.5 RPTT. More specifically, almost half of the *Nematoda* carriage of St_IT2 insects could be ascribed to orders that included known parasites of insects, such as *Rhabditida* and *Mermithida*, and the other half to the order *Trichinellida*, which was also represented in the other libraries.

The *S. titanus* sap meal and habitat should explain the presence of the reads assigned to the *Streptophyta* (plants) transcripts in all libraries as well as the identification in the St_CH library of reads that mapped to the almost complete genome of Tomato mosaic virus (ToMV), which is known to infect grapevines (Martelli and Boudon-Padieu, 2006). *Kitrinoviricota* were also represented by the reads mapping to other Grapevine viruses, such as Grapevine leafroll-associated virus 3 and Grapevine satellite virus in the St_IT2 library, Grapevine asteroid mosaic-associated virus in St_IT1 and St_CH libraries, and finally, Grapevine Syrah virus 1 and Grapevine rupestris vein feathering virus in the St_HU library.

Surprisingly, all libraries showed reads that mapped to transcripts assigned to *Chordata*, mainly to bony fishes, amphibians, and birds, with the highest levels reached in both Italian libraries (an average of 23.5 RPTT). If the presence of all the previous phyla within *S. titanus* metatranscriptome could be considered as conceivable, the identification of high amounts of reads assigned to *Chordata* in an insect that does not feed on vertebrate tissues was worthy of further investigations. The most obvious explanation was that unwanted contamination had occurred at some point during sample manipulations. Although this hypothesis cannot be excluded, a Batch CD-Search against the NCBI's Conserved Domain Database revealed that in both libraries, the most highly expressed *Chordata* transcripts coded for proteins with domains that are very well conserved across different eukaryotic phyla, such as those found in histones, ubiquitins, lectins, zinc-finger proteins, tubulins, and reverse transcriptases (Supplementary Data Sheet 2). In addition, a more thorough blastx analysis showed that for almost all such transcripts, among the first 20 significant hits with similar E-values, there were several proteins belonging to the eukaryotic phyla other than *Chordata*, including *Arthropoda* (Supplementary Data Sheet 3). Therefore, our alternative hypothesis is that at least some of those sequences, and their corresponding reads, could belong to the eukaryotes other than the *Chordata*, possibly to *S. titanus*.

### Phylogenetic Analysis of the *Ophiocordyceps-*Allied Fungus of *S. titanus*

**Figure 1:**
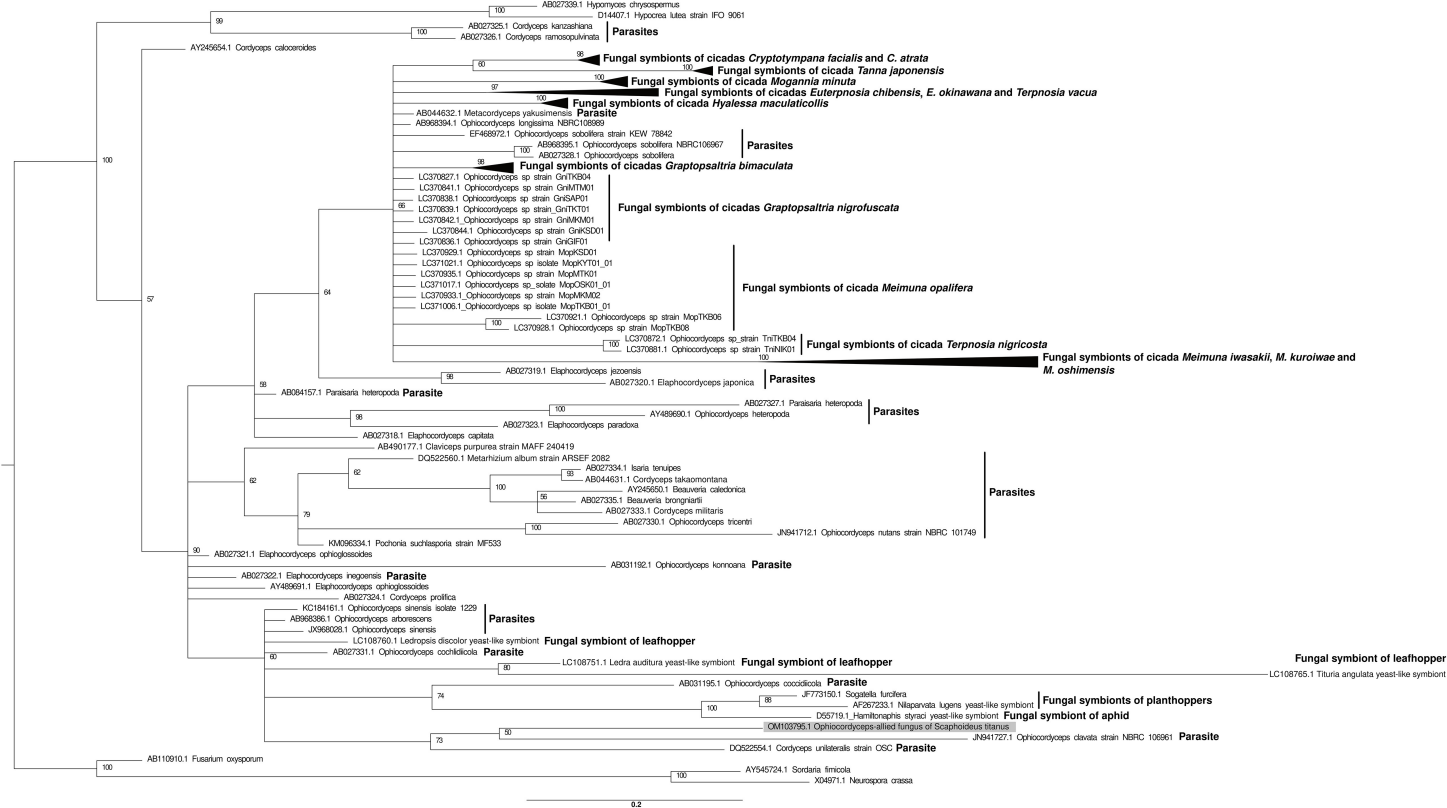
Phylogenetic relationships of the *Ophiocordyceps-*allied fungus of *S. titanus*. A Bayesian phylogeny with 10^7^ generations based on the *Hypocreales* 18S rRNA gene sequences (provided by Matsuura et al., 2018) is shown. The sequences from *Neurospora crassa* and *Sordaria fimicola* (both belonging to the order Sordariales) were used as outgroups. The *Ophiocordyceps-*allied fungus of *S. titanus* is highlighted in a gray box. The collapsed branch named “Fungal symbionts of the cicadas *Graptopsaltria bimaculata* and *G. nigrofuscata”* grouped 11 sequences: LC370827.1 Ophiocordyceps sp strain GniTKB04, LC370836.1 Ophiocordyceps sp strain GniGIF01, LC370841.1 Ophiocordyceps sp strain GniMTM01, LC370839.1 Ophiocordyceps sp strain GniTKT01, LC370842.1 Ophiocordyceps sp strain GniMKM01, LC370838.1 Ophiocordyceps sp strain GniSAP01, LC370844.1 Ophiocordyceps sp strain GniKSD01, LC370858.1 Ophiocordyceps sp strain GbiAMM01, LC370850.1 Ophiocordyceps sp strain GbiNNJ01, LC370857.1 Ophiocordyceps sp strain GbiTKN01, LC370859.1 Ophiocordyceps sp strain GbiOGM01. The collapsed branch named “Fungal symbionts of the cicada *Hyalessa maculaticollis”* grouped five sequences: LC370989.1 Ophiocordyceps sp strain HmaMTK01, LC370983.1 Ophiocordyceps sp strain HmaTKB05, LC370991.1 Ophiocordyceps sp strain HmaMTM01, LC370994.1 Ophiocordyceps sp strain HmaOTR01, LC370993.1 Ophiocordyceps sp strain HmaSap01. The collapsed branch named “Fungal symbionts of the cicada *Mogannia minuta”* grouped three sequences: LC370996.1 Ophiocordyceps sp strain MmiIRO03, LC370997.1 Ophiocordyceps sp strain MmiKom01, LC370999.1 Ophiocordyceps sp strain MmiNNJ01. The collapsed branch named “Fungal symbionts of the cicada *Cryptotympana facialis”* grouped 10 sequences: LC370788.1 Ophiocordyceps sp strain CfaKSD05, LC370815.1 Ophiocordyceps sp strain CfaKWS02, LC370813.1 Ophiocordyceps sp strain CfaISG01, LC370809.1 Ophiocordyceps sp strain CfaNAH01, LC370796.1 Ophiocordyceps sp strain CfaOCP02, LC370806.1 Ophiocordyceps sp strain CfaTKT01, LC370811.1 Ophiocordyceps sp strain CfaISG01, LC370816.1 Ophiocordyceps sp strain CatKNZ01, LC370803.1 Ophiocordyceps sp strain CfaOKZ01, LC370825.1 Ophiocordyceps sp strain CatKNZ0. The collapsed branch named “Fungal symbionts of the cicadas *Euterpnosia chibensis, E. okinawana* and *Terpnosia vacua”* grouped four sequences: LC370894.1 Ophiocordyceps sp strain EokKNG01, LC370900.1 Ophiocordyceps sp strain EokKNG02, LC370888.1 Ophiocordyceps sp strain EUTCHI, LC370866.1 Ophiocordyceps sp strain TERVAC. The collapsed branch named “Fungal symbionts of the cicada *Tanna japonensis”* grouped three sequences: LC370902.1 Ophiocordyceps sp strain TjaTKB04, LC370916.1 Ophiocordyceps sp strain TjaSMR02, LC370909.1 Ophiocordyceps sp strain TjaYIT01. The collapsed branch named “Fungal symbionts of the cicadas *Meimuna iwasakii, M. kuroiwae* and *M. oshimensis”* grouped seven sequences: LC370967.1 Ophiocordyceps sp strain MiwITN01, LC370973.1 Ophiocordyceps sp strain MiwYRB01, LC370960.1 Ophiocordyceps sp strain MkuYGJ01, LC370953.1 Ophiocordyceps sp strain MosTKN02, LC370947.1 Ophiocordyceps sp strain MosTKN01, LC370940.1 Ophiocordyceps sp strain MosKNG01, LC370946.1 Ophiocordyceps sp strain MosKNG02. “Parasite” refers to known entomopathogens. Only the posterior probability values higher than 50% are shown. The scale bar refers to a phylogenetic distance of 0.2 nucleotide substitutions per site.

Even though the libraries were rRNA-depleted before sequencing, rRNA reads were still removed during the quality filtering process of the six libraries. Taking advantage of such discarded reads, it was possible to assemble partial sequences of the same fungal 18S rRNA gene in all libraries. The blastn analysis of the longest (550 bp, retrieved from the St_CH library) showed 95.6% identity and 100% query coverage with *Ophiocordyceps sinensis* isolate 1229 28S-18S ribosomal RNA intergenic spacer, partial sequence (GenBank Accession: KC184161.1). The sequence was aligned to the ones used in the work of Matsuura et al. (2018) to infer the phylogeny of fungal symbionts of cicadas. The tree topology (Figure 1) showed that the *Ophiocordyceps*-allied fungus of *S. titanus* had a distant evolutionary connection with yeast-like symbionts of other leafhoppers, planthoppers, and one species of aphid and a closer relatedness with the entomopathogens of the *Ophiocordycipitaceae* family.

### “Dark Matter” Matters: Recovering Segment 12 of Scaphoideus Titanus Reo-Like Virus 1

The transcriptional “dark matter” potentially hides the biological information that cannot be interpreted by current methods. A full elucidation of the role of all the *S. titanus* “unknowns” is likely to be extremely time-consuming. However, the expression levels combined with co-occurrence analysis across libraries could be one of the criteria that might help prioritize the relevant transcripts for further investigation efforts. Focusing only on the putative protein-coding transcripts at least 1,000 nt-long (Supplementary Table 1), we tried to identify segment 12 of the Scaphoideus titanus reo-like virus 1, which was missing from the previous analysis (Ottati et al., 2020). This virus was known to be present only in the St_USA library. A total of 256 out of 700 protein-coding transcripts of the St_USA library were found in at least one of the other libraries, so they were excluded from further analysis. Then, the reads were mapped to the remaining contigs taking into account the orientation, that is, whether they mapped in sense or antisense orientation. In the end, only one transcript presented an amount of reads mapping to both strands within the range found for the previously identified 11 segments of the virus (Table 3). The alignment of the corresponding putative protein with segment 12 of the Homalodisca vitripennis reo-like virus (GenBank accession ACO37244.1) did show that the missing viral segment could not have been retrieved by a simple sequence similarity search (Supplementary Image 3).

**Table 3:** Reads that mapped to the 12 segments of Scaphoideus titanus reo-like virus 1 in sense or antisense orientation.

**Scaphoideus titanus reo-like virus 1 segments**	**Sense**	**Antisense**
QIJ56927.1 minor core protein	104,786	13,800
QIJ56933.1 non-structural protein	100,248	22,966
QIJ56931.1 major core protein	285,976	87,244
QIJ56926.1 non-structural protein	170,378	9,066
QIJ56928.1 minor core protein	406,038	67,648
QIJ56932.1 zinc-finger protein	558,732	20,090
QIJ56924.1 RNA-directed RNA polymerase	1,151,874	472,182
QIJ56929.1 RNA-binding protein	1,135,154	59,232
QIJ56930.1 capsid protein	136,912	5,544
QIJ56934.1 non-structural protein	173,620	21,580
QIJ56925.1 non-structural protein	122,914	8,190
OM103795 non-structural protein (putative segment 12)	558,494	10,524

*The newly identified segment (GenBank: OM103795) is reported in the last row*.

## Discussion

The RNA-Seq datasets previously used for insect virus discovery in the phytoplasma vector *S. titanus* were re-analyzed in this work to generate the *de novo* transcriptome assembly of the insect host and extrapolate qualitative information regarding the composition of its associated microbiome in different geographical areas. The Omics techniques have completely removed the limitations and boundaries associated with the studies of individual species in the laboratory, thus, significantly advancing our in-depth knowledge of the entire microbial communities in their natural ecosystems.

The comparisons of taxonomic abundance across libraries were kept to a minimum because mixed-species RNA-Seq data capture both the transcriptional expression levels and species abundance. Therefore, it is difficult to assess whether higher amounts of reads assigned to a taxon could stem from either higher expression levels per cell (the taxon is present in low amounts, but it is transcriptionally very active) or a higher nucleus count (the taxon is present in high amounts, but its transcriptional activity is low).

The newly assembled *S. titanus* transcriptome, alongside the transcriptome of the other phytoplasma vector *Euscelidius variegatus* (Galetto et al., 2018), provides a valuable sequence resource for downstream applications, such as the implementation of strategies based on RNA interference (RNAi) for studying the insect gene function as well as for devising new biocontrol tactics against the insect vector (Ripamonti et al., 2022). Analogously, the analysis of the composition of *S. titanus* microbiome and a better understanding of the complex interactions within the holobiont could further contribute to the identification of novel targets for sustainable strategies to counteract the Flavescence dorée spread in Europe.

The paucity of genomic information about *S. titanus* and the fact that samples were caught in the wild often made the assignment of transcripts to a specific taxon quite challenging, with high percentages of ambiguities or no similarities found. Therefore, instead of assigning reads and sequences to species, we have mostly preferred assignments to high taxonomic ranks, such as phyla, and, only in some specific cases, to families. The exception is represented by transcripts belonging to some insect endosymbionts, whose transcript abundance within the phylum *Bacteroidetes* made taxonomic assignments unambiguous even at the genus and species levels.

Apart from the viral component, which was extensively described in previous work (Ottati et al., 2020), the most interesting aspect of *S. titanus* microbiome is its bacterial diversity, which, at least theoretically, could represent the most promising target for pest management.

### Identifying *S. titanus* Primary and Secondary Symbionts

Most sap-feeding insects of the suborder Auchenorrhyncha host two symbionts: *Ca*. Sulcia muelleri (*Bacteroidetes*), also referred to as “the primary symbiont,” and usually a proteobacterium as a co-primary symbiont, such as *Ca*. Nasuia deltocephalinicola in *Deltocephalinae* leafhoppers (Noda et al., 2012), *Ca*. Vidania fulgoroideae in planthoppers (Gonella et al., 2011), *Ca*. Zinderia insecticola in spittlebugs (McCutcheon and Moran, 2010), *Baumannia cicadellinicola* in sharpshooter leafhoppers (Wu et al., 2006), and *Hodgkinia cicadicola* in most cicadas (McCutcheon et al., 2009).

The presence of *Ca*. Sulcia muelleri in all our libraries was unexpected since up to now, *S. titanus* was supposed to lack this primary symbiont (Bennett and Moran, 2013). Conversely, none of the aforementioned co-primary symbionts was identified among the *Proteobacteria*, which, unlike *Bacteroidetes*, were represented by a striking variety of species in all libraries. This high degree of biodiversity is as per the microscopic observations of *S. titanus* midgut, which revealed the existence of a very polymorphic bacterial flora (Crotti et al., 2009). *Wolbachia* and *Rickettsia*, which are strikingly widespread in various insect hosts (Perlman et al., 2006; Correa and Ballard, 2016), were also present within the *S. titanus* microbiome but represented by a low number of reads in all libraries.

Previous ultrastructural observations have shown that *Cardinium* sp. (*Bacteroidetes*) and yeast-like endosymbionts (YLSs) belonging to *Sordariomycetes* (Marzorati et al., 2006; Sacchi et al., 2008) are harbored by *S. titanus* and are transovarially transmitted to the offspring. The reads assigned to the family *Ophiocordycipitaceae* (class *Sordariomycete*s) were present in all our libraries, whereas the sequences assigned to *Cardinium* were present only in the European libraries. Interestingly, the specific oligonucleotide probe that Sacchi et al. (2008) designed on the small subunit rRNA sequence to localize the yeast endosymbiont through *in situ* hybridization is contained in the 18S rRNA gene region of the *Ophiocordyceps-*allied fungus identified in this work (data not shown). Therefore, it is very likely that the transcripts and reads assigned to the family *Ophiocordycipitaceae* in all our libraries belonged to *S. titanus* YLSs that were experimentally characterized. In this context, it is notable that also fungal endosymbionts of cicadas (Matsuura et al., 2018), leafhoppers (Nishino et al., 2016; Kobiałka et al., 2018), aphids (Vogel and Moran, 2013), scale insects (Podsiadło et al., 2018; Szklarzewicz et al., 2021), and planthoppers (Suh et al., 2001) appeared to be phylogenetically related to the genus *Ophiocordyceps*.

The primary symbionts such as *Ca*. Sulcia muelleri can provide their insect hosts with essential amino acids, vitamins, and many cofactors that are usually present in very low concentrations in their diet. By contrast, *Cardinium* is generally considered as a “secondary symbiont,” which is a facultative bacterial endosymbiont not essential for insect survival. Despite that, a long holobiont coevolution in the nutritional insect symbioses might transform the facultative symbionts into primary symbionts, as occurred, for example, in the symbiont *Candidatus* Serratia symbiotica in the cedar aphid *Cinara cedri* (Gosalbes et al., 2008).

Presuming that the co-primary symbiont is missing from the *S. titanus* microbiome, its role might be assumed by the yeast-like endosymbiont, as it was already hypothesized for other *Hemiptera* (Matsuura et al., 2018). The metabolic pathways of such fungal symbionts are supposed to compensate for the absence of the co-primary symbionts. *Ca*. Sulcia muelleri, in fact, typically produces eight out of the 10 amino acids essential for animals, but it is unable to produce histidine or methionine, which are usually provisioned by the co-primary symbiont (reviewed by McCutcheon and Moran, 2010). The fungal symbiont genome of the cicada *Meimuna opalifera*, for example, retained the genes for the synthesis of these two amino acids (Matsuura et al., 2018). Taking advantage of the fact that the metatranscriptomic approaches also provide information about which genes the microbial community is expressing, in the two Italian libraries it was possible to identify the same *Ophiocordycipitaceae* transcript (TRINITY_DN94655_c1_g3_i1 in St_IT1 and TRINITY_DN76594_c0_g1_i1 in St_IT2) coding for methionine synthase II (cobalamin-independent), one of the key enzymes in the methionine biosynthesis. Finally, the *Ophiocordyceps-*allied fungus of *S. titanus* appeared to be only distantly related to other taxonomically characterized fungi and, therefore, might represent a member of a new fungal species.

### Other Insect-Associated Microorganisms

Besides the microorganisms that are transmitted host-to-host, insect microbiomes are populated by the taxa acquired from the outside environment and appear to colonize the hosts opportunistically. Diet, life stage, and the local environment are highly significant factors explaining the structuring of the microbial community composition (Gurung et al., 2019). Although the 16S rRNA sequences ascribed to the genus *Asaia* (*Alphaproteobacteria*) were previously identified in the *S. titanus* microbiome (Marzorati et al., 2006), the metatranscriptomic data could associate very few reads to the class *Alphaproteobacteria* and none specific to this genus. Therefore, its presence may not be stable and probably restricted to specific environmental conditions.

Part of the differences between the American specimens and the European ones could be explained by the environment *S. titanus* usually lives in. In Europe, it is abundant in the vineyard agroecosystems, whereas in the USA, it is mostly found in unmanaged open woodlands. Its feeding habits, for example, justify the presence of the reads assigned to *Streptophyta* in all libraries, but its adaptation to feed and breed mainly on the *Vitis* species in Europe determined the identification of *Vitis*-related viruses only in the European libraries. Furthermore, the presence of reads belonging to the phytoplasmas (*Tenericutes*) becomes of paramount importance from both epidemiological and economic viewpoints, because only in Europe *S. titanus* acquires and transmits Flavescence dorée phytoplasma. Notably, the phytoplasma transcripts were identified only in one of the Italian libraries (St_IT1), even though FDp spread to the vineyards in France, Hungary, and Switzerland (EFSA Panel on Plant Health et al., 2016). This result reaffirms that in natural conditions, the microbial communities that are not stably associated with their insect hosts are influenced by a variety of environmental factors. The reads assigned to *Tenericutes* appeared to belong mainly to “Candidatus phytoplasma ziziphi,” even if this phytoplasma has never been reported in Europe (CABI, 2022, https://www.cabi.org/isc/datasheet/118192). The sensitivity of metatranscriptomics depends crucially on the comprehensiveness of public databases. At the time of writing this manuscript, although in the NCBI nr database there were hundreds of sequences belonging to FDp, they stemmed from only 35 different genes. Therefore, the automatic pipeline predominantly assigned transcripts and reads to “Candidatus phytoplasma ziziphi,” whose genome is completely sequenced and publicly available. Interestingly, “Candidatus phytoplasma ziziphi” belongs to the 16SrV phylogenetic group, the same as FDp.

The Zooplankton include a diverse taxa, many of which are microscopic: *Rotifera*, for example, are generally 50–2,000 μm long. They are ubiquitous in aquatic environments, so their presence in association to *S. titanu*s can be explained by an environmental exposure retained on the insect surface.

The nematode-insect associations are ubiquitous and range from parasitic or entomopathogenic to simply phoretic (nematodes vectored from host-to-host by insects). The St_IT2 library is characterized by an over-representation of reads assigned to the *Nematoda*, half of which to the orders *Rhabditida* and *Mermithida*, which were almost absent from the other libraries. Based on the fact that some nematode parasites of the insects belong to these two orders (Capinera et al., 2008), we can reasonably speculate that at least part of the insects pooled in library St_IT2 was parasitized, whereas insects from the other libraries probably phoretically carried microscopic nematodes, mostly belonging to the order *Trichinellida*.

### Ambiguous Assignments and Dark Matter in the Context of a Missing Genome Reference

From 0.4 to 1.6 million reads for each library mapped to taxonomically ambiguous contigs. In 2020, it was estimated that around 7.6% of the total number of sequences deposited in the NCBI nr database are potentially taxonomically misclassified (Bagheri et al., 2020). This percentage is not negligible, because the error is likely to be propagated by tools that are based on homology to the existing sequences to predict the taxonomic assignments. In our case, even if we assigned transcripts and reads to the lowest common ancestors, most of the ambiguities were between the insect host (a eukaryote) and its bacterial components, rather than between two microorganisms (data not shown). These uncertainties are probably caused by high-throughput sequencing studies that wrongfully assigned sequences to the host instead of the associated microorganisms or vice versa.

Although the reads assigned to the *Chordata* passed stringent taxonomic filtering, their presence in association with an insect that does not feed on the tissues of vertebrates suggested, as the most immediate explanation, the accidental presence of contaminating nucleic acids, even if the *in-silico* analysis includes the removal of reads and transcripts belonging to the most frequent contaminants, namely human, dog, mouse, and cat. By contrast, the small range of highly conserved domains identified in the transcripts assigned to the *Chordata* supports the alternative hypothesis that at least part of these sequences might belong to other phyla, *Arthropoda* included. True contamination, especially in the Italian libraries where the *Chordata* read counts are the highest, would have reasonably implied the presence of mammal transcripts encompassing a much broader range of gene functions, including typical housekeeping genes, such as ribosomal proteins, tRNA ligases, or transcriptional factors, which instead are completely missing. The genome sequencing of *S. titanus* will clarify the origins of such transcripts and reads.

Besides the taxonomic ambiguities, the most intriguing transcripts are those with predicted complete open reading frames that lack any detectable similarities to the previously published sequences. Such transcripts could represent a gold mine of new genes and functions that deserve a closer look. Given the great genetic variability observed in the viruses, the discovery of novel viral genomes or missing segments within the dark matter has several precedents in the literature (Chiapello et al., 2020; Obbard et al., 2020; Sutela et al., 2020; Batson et al., 2021; Forgia et al., 2021). In our work, we focused attention on a missing segment of a reo-like virus that was known to be present only in the American library. However, the prioritization criteria for further investigations into the dark matter could have been the opposite, that is, considering unknown transcripts that occurred in all libraries. In this case, these might represent novel uncharacterized genes expressed either by the insect or by the microorganisms ubiquitously associated with the host.

## Conclusions

In conclusion, these data have considerably expanded our understanding of the microbial diversity associated with the *S. titanus* populations from different geographical locations. Although the prevalence of every single microbial component is still to be assessed by testing individual insects, the core *S. titanus* microbiome of the six libraries comprises *Ca*. Sulcia muelleri and an *Ophiocordyceps*-allied fungus, are both maternally transmitted and, as such, form a stable and permanent association with the insect. Only in the European libraries, *Cardinium* sp. has been identified as another dominant member of the microbiome. The high transcriptional activity of these three components suggests that they could significantly impact the dynamics of the insect microbiota in the European populations. As a consequence, they are likely to influence the acquisition, maintenance, and transmission of *S. titanus*-borne FDp. The fungal symbionts, in particular, might represent a selective target to reduce the *S. titanus* presence in the European vineyards. Fungicides have proven effective under controlled conditions against the YLS species harbored by two planthoppers, *Nilaparvata lugens* (Shentu et al., 2016) and *Sogatella furcifera* (Pang et al., 2020), even if they are not environmental-friendly alternatives to insecticides. The *Cardinium* species, besides being known to negatively influence the reproduction of their hosts (Hunter et al., 2003; Weeks et al., 2007), might be also used for paratransgenesis approaches, namely as vehicles to specifically express foreign traits that may interfere with the phytoplasma acquisition/transmission or insect fitness. Paratransgenesis, for example, was successfully used against another agricultural pest, *Homalodisca vitripennis*, to block the transmission of the bacterial pathogen *Xylella fastidiosa* to the grape plants (Arora et al., 2018).

The other components of the *S. titanus* microbiome appeared to be intermittent or restricted to specific environmental conditions, but their presence and interactions should be taken into account when designing targeted biocontrol interventions that aim at the perturbation of the existing microbial communities (Kessell et al., 2020).

## Data Availability Statement

The datasets presented in this study can be found in online repositories. The names of the repository/repositories and accession number(s) can be found in the article/Supplementary Material.

## Author Contributions

SA conceived and designed the study and wrote the first draft of the manuscript. SA, MR, and MV contributed to the data analyses. SA, MR, MV, LG, CM, and MT critically revised the manuscript. CM and MT acquired the funding. All authors read and approved the submitted version.

## Funding

This project has received funding from the European Union's Horizon 2020 research and innovation program under grant agreement no. 773567. This research was also supported by Premio per Progetti di Ricerca 2021 DISBA-CNR, Project Phaser.

## Conflict of Interest

The authors declare that the research was conducted in the absence of any commercial or financial relationships that could be construed as a potential conflict of interest.

## Publisher's Note

All claims expressed in this article are solely those of the authors and do not necessarily represent those of their affiliated organizations, or those of the publisher, the editors and the reviewers. Any product that may be evaluated in this article, or claim that may be made by its manufacturer, is not guaranteed or endorsed by the publisher.
