# Supplementary material for: Metatranscriptomic Assessment of the Microbial Community Associated With the Flavescence dorée Phytoplasma Insect Vector Scaphoideus titanus
Source: Front Microbiol. 2022 Apr 19;13:866523. doi: 10.3389/fmicb.2022.866523 (PMC9063733; doi:10.3389/fmicb.2022.866523)
Supplement: Supplementary file 1 [file Table_1.pdf]

Supplementary Table 1 Number of expressed transcripts without any detectable homologs to previously published sequences.

	St_IT1	St_IT2	St_FR	St_CH	St_HU	St_USA
**Transcripts ≥ 1000 bp**	9089	5569	4337	7001	10091	4031
**With at least one predicted ORF (TransDecoder)**	1122	644	545	726	1011	700
