# Supplementary material for: Metatranscriptomic Assessment of the Microbial Community Associated With the Flavescence dorée Phytoplasma Insect Vector Scaphoideus titanus
Source: Front Microbiol. 2022 Apr 19;13:866523. doi: 10.3389/fmicb.2022.866523 (PMC9063733; doi:10.3389/fmicb.2022.866523)
Supplement: Supplementary file 5 [file Image_1.pdf]

St_IT1 microbiome composition

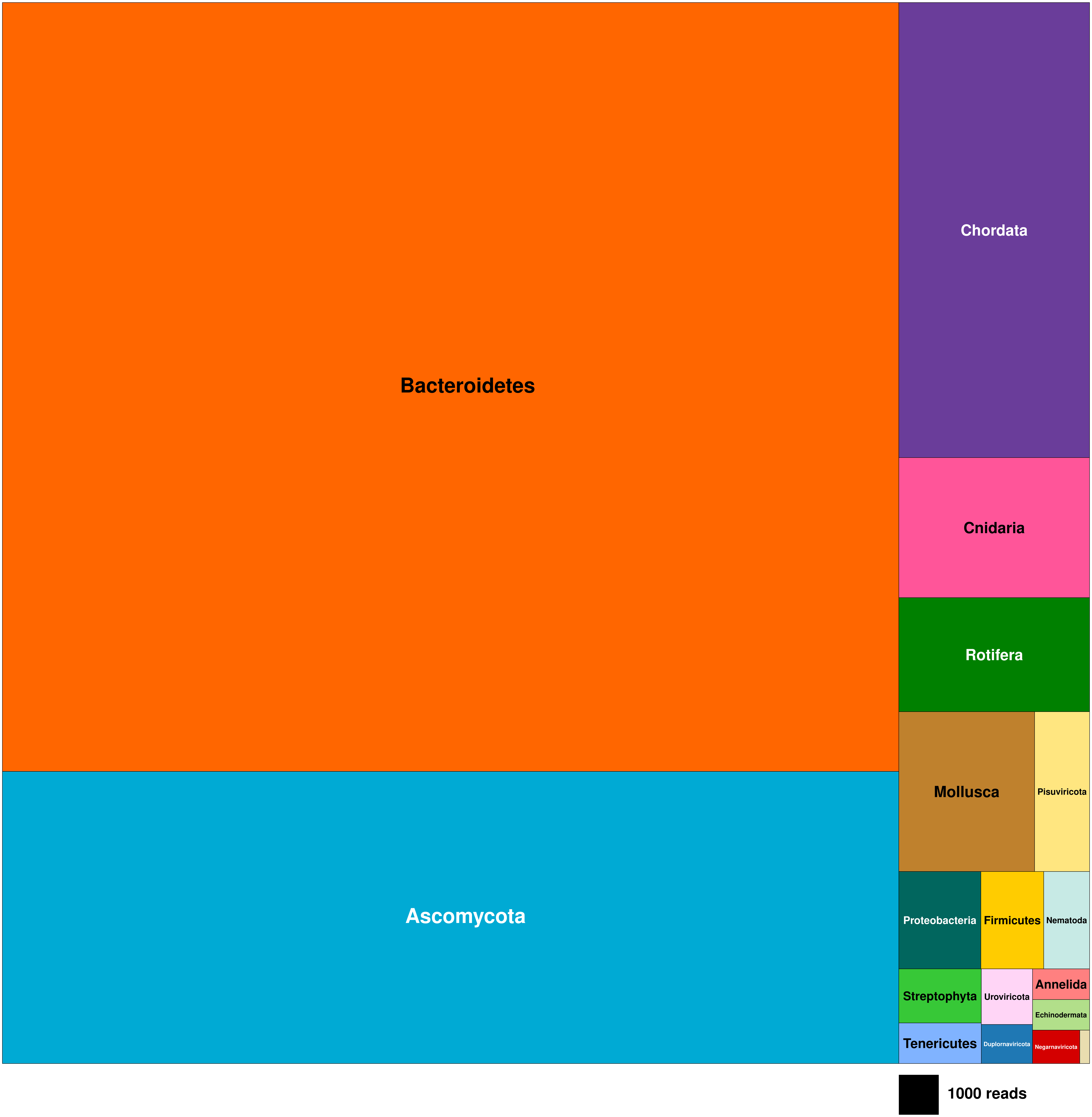

St_IT2 microbiome composition

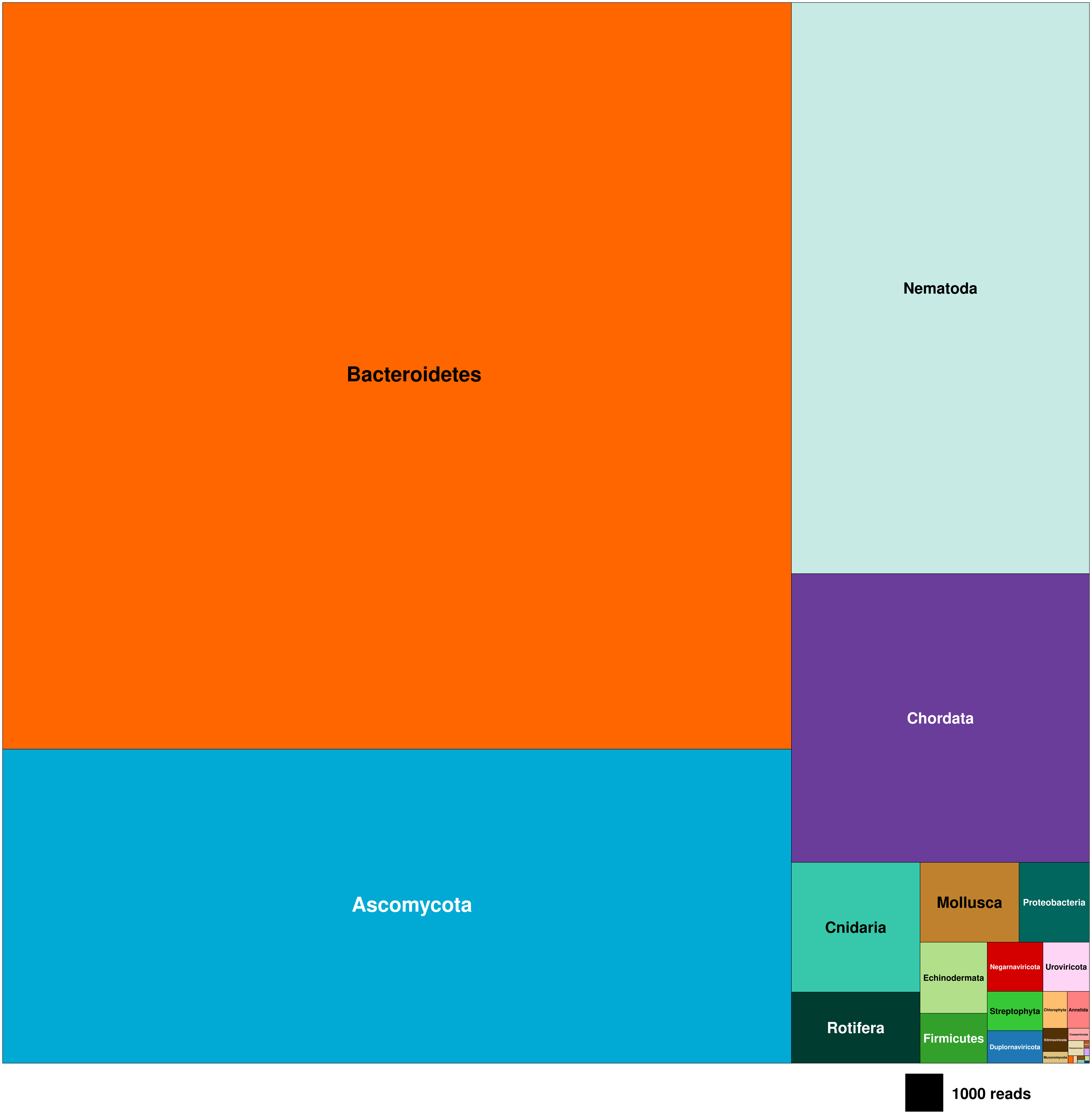

St_FR microbiome composition

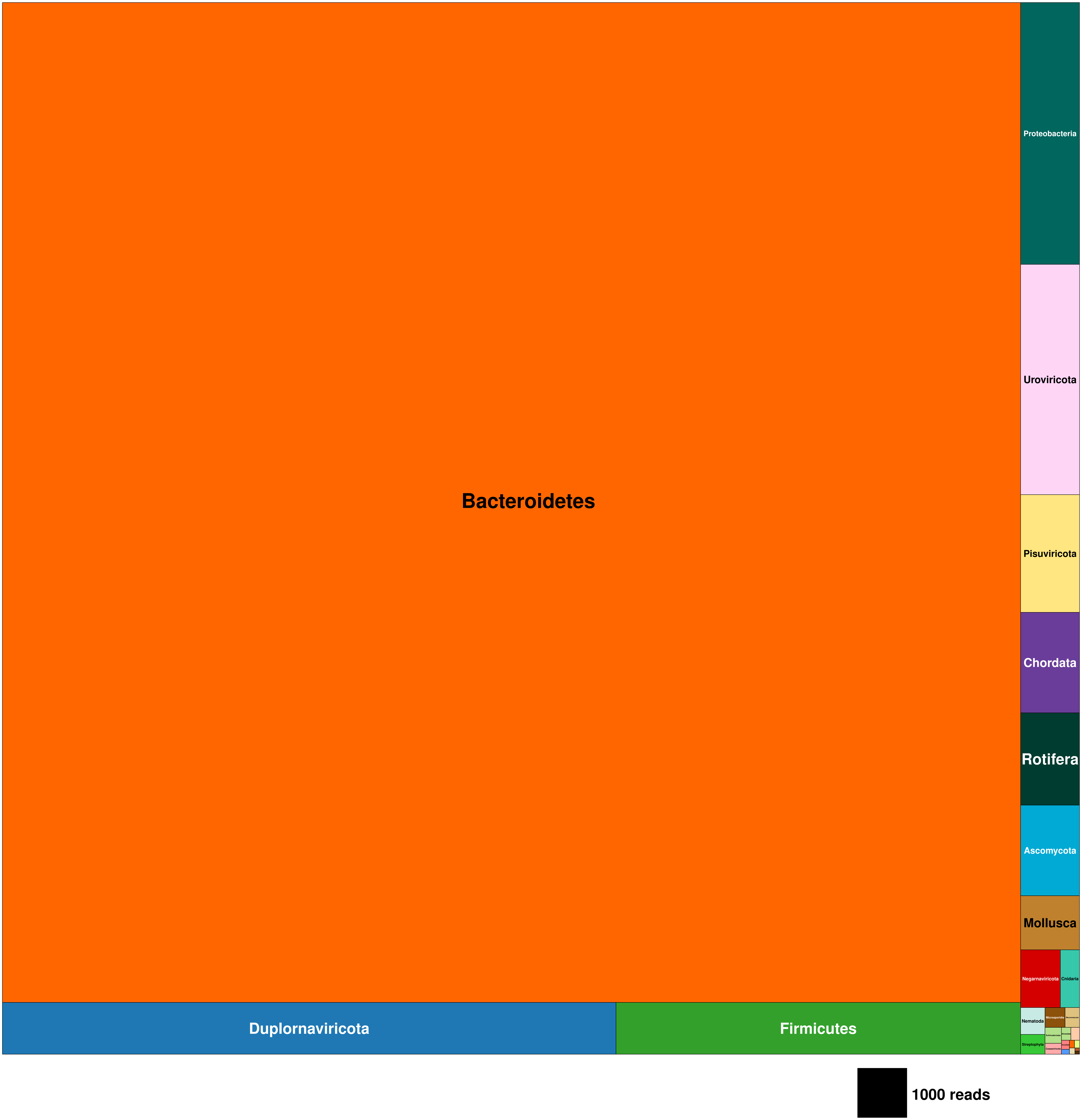

St_CH microbiome composition

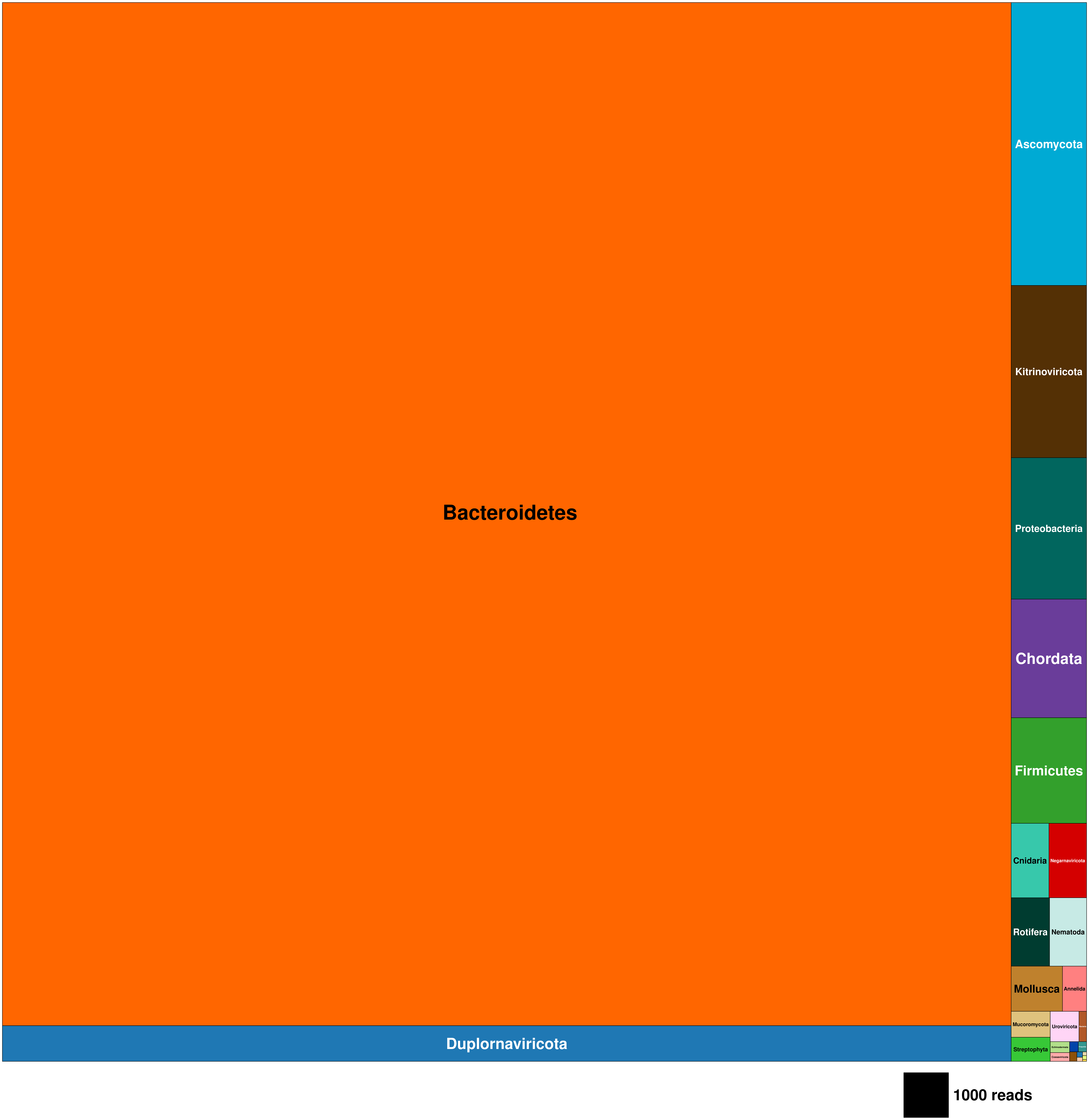

St_HU microbiome composition

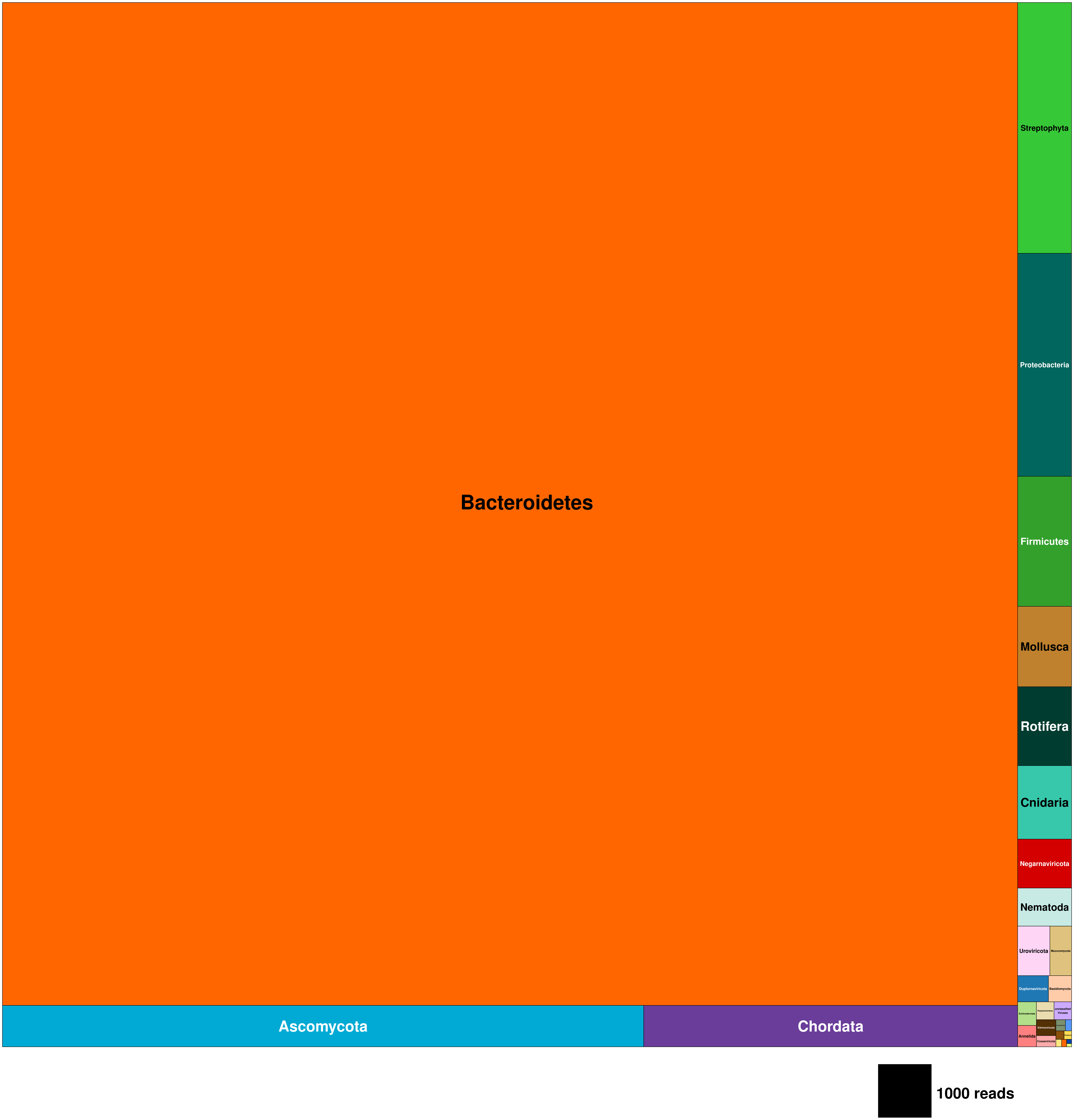

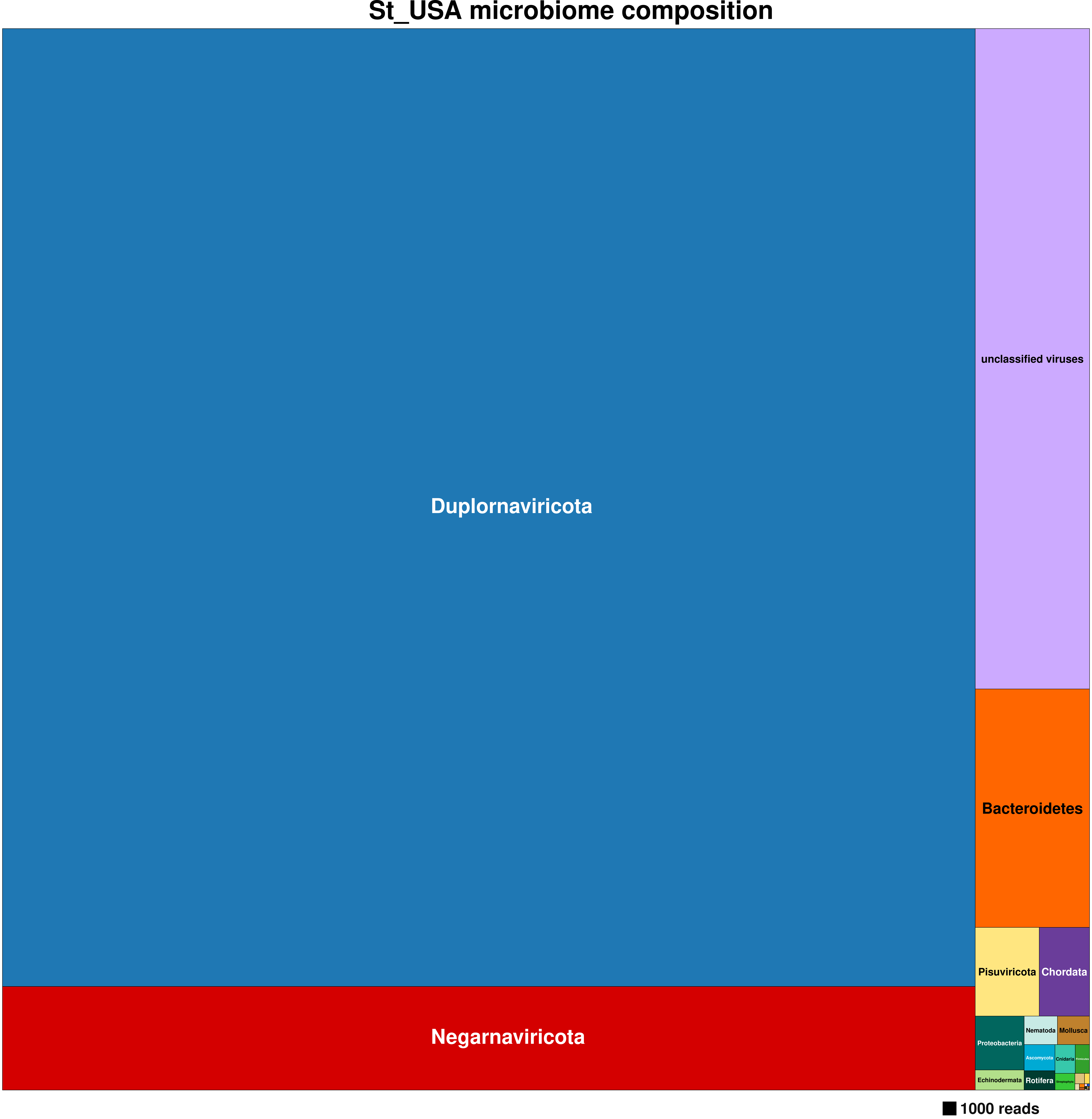

Figure S1 Quantitative treemap overviews of how the assembled non-host reads mapped across the viral, prokaryotic, and eukaryotic phyla in the six libraries.

The block areas are plotted in proportion to the number of reads assembled into contigs that could be assigned to a given phylum**.**
