# Supplementary material for: Metatranscriptomic Assessment of the Microbial Community Associated With the Flavescence dorée Phytoplasma Insect Vector Scaphoideus titanus
Source: Front Microbiol. 2022 Apr 19;13:866523. doi: 10.3389/fmicb.2022.866523 (PMC9063733; doi:10.3389/fmicb.2022.866523)
Supplement: Supplementary file 6 [file Image_2.pdf]

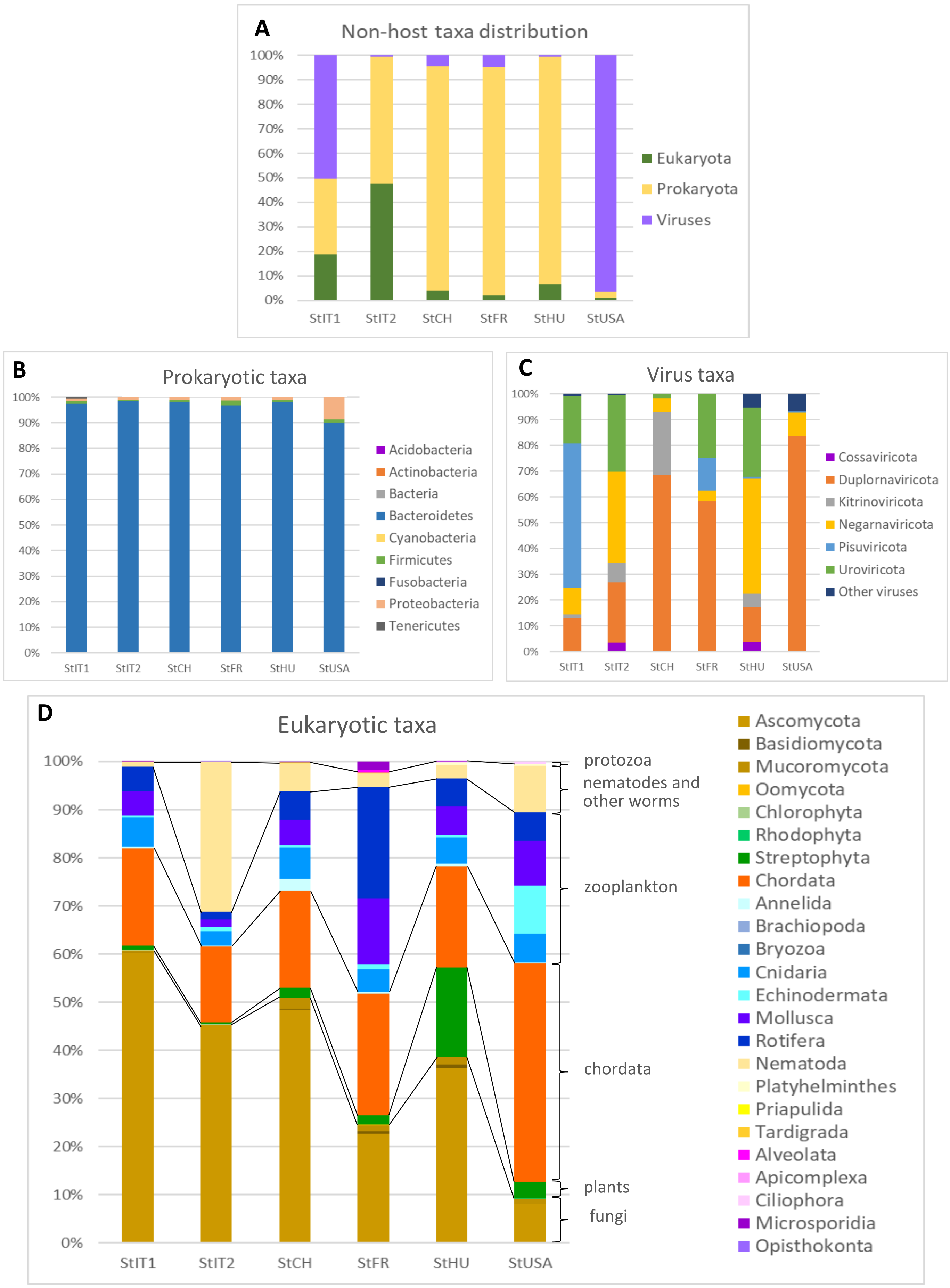

Figure S2 Percentage of reads assigned to different taxa in the six libraries. Plotted bars report an overview of non-host reads assigned to viral, bacterial, and eukaryotic taxa (**A**) and the details for bacterial (**B**), viral (**C**) and eukaryotic (**D**) taxa. Taxa are color-coded as indicated in the legend (right of each graph).
