# Supplementary material for: Metatranscriptomic Assessment of the Microbial Community Associated With the Flavescence dorée Phytoplasma Insect Vector Scaphoideus titanus
Source: Front Microbiol. 2022 Apr 19;13:866523. doi: 10.3389/fmicb.2022.866523 (PMC9063733; doi:10.3389/fmicb.2022.866523)
Supplement: Supplementary file 7 [file Image_3.pdf]

```
putative_segment12      MFFDIVGRMVLPAAIRSSRKSADKTKDKELKHREEEVKNKESVDPSNTKFAT-----PPN    55
ACO37244.1              -----MSSVQLPRSKRNTRNTS-------LPKT-SVAQPTEKLAPDATRNTNASGGASIT    47
                             :. : ** : *.:*:::       * :  . .: .*.: *. *: :.       .

putative_segment12      LISQGIIS---TETISYQKLENNLAHGQFPPENCQPLQQREKRGYRGSTPQSDGAPSHTI    112
ACO37244.1              SITTGPVTTVTNKLFKPEFLTNNICNGLFESDMP-EMVENDGQGSGGVTPEPISNASSDG    106
                         *: * ::   .: :. : * **:.:* *  :    : :.: :*  * **:  .  *

putative_segment12      EGVAISVQDSRQSNVRDDLNTGRRDELILERGPTSDVISKL-------------SPFNRL    159
ACO37244.1              KSFSLCESHSEQSSIRDSVDTGRGNVQVHERDGSNYNANGVVGNDQYGSSILSDREFSSV    166
                        :..::. ..*.**.:**.::*** :  : **. :.   . :               *. :

putative_segment12      HLYQYSELIELNPLTVL--------KEVTEHVDLMQQGFSLQCLLTNLATYNTMRMSYSD    211
ACO37244.1              TL---SAMMGLSGVDCFIDCMKMVVRVVNDRNELLNATNHI----TT-----MLAMSHSG    214
                         *   * :: *. :  :        : *.:: :*::    :    *.      : **:*.

putative_segment12      KTIKSWSLTSVILRNYLSLVGCLILHKISSAVGDDDKKTIIDELQSANITVEE--SCVHF    269
ACO37244.1              MCATKITLHSFILAGLKH-FFCALTKVLKCDKH--DEGVL----PTENYSYDKSNDVMTF    267
                           .. :* *.** .    . * : : :..     *: .:     : * : ::  . : *

putative_segment12      SFK--SIVNDDIKTTTIMTRPGSLPDASD-DTMH-GICWF------- 305
ACO37244.1              NLRIFSFSTNEYSTIRILVKDDIFCSLKNEDVLLLLNCIGIDTHETR 314
                        .::  *: .:: .*  *:.: . : . .: *.:    *
```

Figure S3 Alignment of the putative segment 12 of Scaphoideus titanus reo-like virus 1 to segment 12 of Homalodisca vitripennis reo-like virus (GenBank accession ACO37244.1).
